# Genome-resolved metagenomics identifies the particular genetic traits of phosphate-solubilizing bacteria in agricultural soil

**DOI:** 10.1038/s43705-022-00100-z

**Published:** 2022-02-16

**Authors:** Xingjie Wu, Zhenling Cui, Jingjing Peng, Fusuo Zhang, Werner Liesack

**Affiliations:** 1 College of Resources and Environmental Sciences, National Academy of Agriculture Green Development, Key Laboratory of Plant-Soil Interactions, Ministry of Education, China Agricultural University, Beijing, China; 2 Research Group “Methanotrophic Bacteria and Environmental Genomics/Transcriptomics”, Max Planck Institute for Terrestrial Microbiology, Marburg, Germany

**Keywords:** Soil microbiology, Next-generation sequencing, Microbial ecology, Biogeochemistry

## Abstract

Bacteria play a key role in phosphate solubilization, but related genome-centric research on agricultural microbiomes is scarce. Here, we reconstructed 472 metagenome-assembled genomes (MAGs) covering agricultural soils from six long-term field trials across China. A total of 79 MAGs contained *gcd* encoding quinoprotein glucose dehydrogenase (GCD), which is the key biomarker for phosphate solubilization. Our findings showed that all GCD-MAGs represent potentially novel species, with *gcd* copy numbers varying from 1 to 10 per genome. Large genome size, a high ratio of glycosyl hydrolase genes, and increased capacity for carbohydrate utilization were specific traits of GCD-MAGs. Notably, the *gcd* copy number showed a significant and positive correlation with genome size. Generated using a machine learning approach, our findings were validated in a dataset of 692 genotypes covering the 18 bacterial families to which the 79 GCD-MAGs belong. Our results improve the knowledge of both the diversity and the genetic composition of phosphate-solubilizing bacteria. In particular, they reveal a genomic link between phosphate solubilization capacity and increased potential for carbohydrate metabolism, which may accelerate targeted engineering and improve management practices for sustainable agriculture.

Phosphorus (P) is one of the most essential elements for all biota to maintain basic metabolic activities and ecosystem functions [1–3]. Inorganic P solubilization and organic P mineralization, as well as cellular phosphorus turnover, make microbial communities central to soil P cycling [3–7]. The *gcd* gene, which encodes quinoprotein glucose dehydrogenase (GCD), has been shown to be the most reliable biomarker for identifying phosphate-solubilizing bacteria (PSB) [4, 7]. PSB take up solubilized phosphate via a phosphate-inorganic transport (Pit) system or a phosphate-specific transport (Pst) system [8, 9]. Despite the importance of PSB for mediating phosphorus limitation in soils, genome-centric metagenomics research assessing phosphate solubilization potential in terrestrial microbiomes is scarce. However, this limited research showed that genome-centric exploration of PSB enables the discovery of novel PSB species and the identification of their genetic potential [4, 7]. Here, we applied metagenomics coupled with machine learning to explore the genome-resolved diversity of PSB among agricultural microbiomes and to identify particular genetic traits significantly enriched in these bacteria.

The topsoils of six long-term fertilizer field trials (>10 years) in China were selected for metagenomic analysis (→ see Supplementary Text for Materials and methods information) (Table S1). Our metagenomic approach yielded 472 metagenome-assembled genomes (MAGs) spanning 21 phyla (Fig. S1). A total of 79 MAGs were identified to harbor *gcd* (GCD-MAGs) (Fig. S2; Table S2), with gene copy numbers ranging from 1 to 10 per genome (Fig. 1a, b; Tables S3, S4). This number greatly exceeds the number of MAGs (36) found to harbor *gcd* among 424 MAGs obtained from reforested postmining soil [7]. In our study, the genetic potential for P solubilization was a specific trait that existed in MAGs affiliated with five phyla: *Acidobacteria*, *Bacteroidetes*, *Chloroflexi*, *Gemmatimonadetes*, and *Proteobacteria*. Collectively, the GCD-MAGs were assigned to 7 classes, 15 orders, 18 families, and 24 genera (Fig. 1a). *Chloroflexi* has not previously known to harbor PSB [10]. None of the GCD-MAGs could be classified at the species level in the GTDB-TK database, thus representing potentially novel species with no close reference genomes (Fig. S1).

**Fig. 1:**
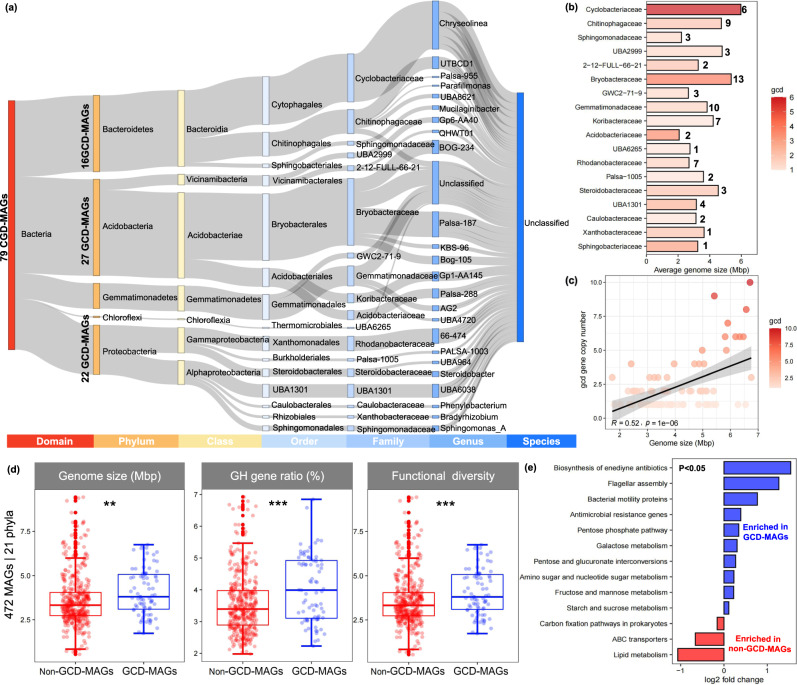
Phylogenetic affiliation, average genome size, and correlation between genome size and *gcd* gene copy number among the 79 GCD-MAGs (particular genes involved in P cycling are shown in Table S3). Phylogenetic affiliation of the 79 GCD-MAGs detected across six agroecosystems in China (**a**). Number of GCD-MAGs, average genome size, and genome-averaged *gcd* copy number of GCD-MAGs affiliated with a particular bacterial family. The number of GCD-MAGs obtained for each family is given in bold. The genome-averaged *gcd* copy number of GCD-MAGs is indicated by color (see scale) (**b**). Significant correlation between genome size and *gcd* gene copy number (Pearson correlation; *P* < 0.001) (**c**). Distribution range of genome size, GH (glycoside hydrolases) gene ratio, and functional diversity between genotypes that contain or do not contain *gcd*. This dataset is composed of the 472 MAGs obtained in this study (**d**). Functional diversity is represented by the Shannon index and was calculated based on 10,799 KEGG-queried genes. Functional traits whose presence significantly (*P* < 0.05) differs between GCD-MAGs and non-GCD-MAGs. The analysis was performed at KEGG level 3 using DESeq2 and involved the 472 MAGs obtained in this study (**e**).

On average, the 79 GCD-MAGs (ranging from 1.73 to 6.75 Mbp) had significantly (*P* < 0.01) larger genomes (4.10 Mbp) than did the 393 non-GCD-MAGs (3.62 Mbp) (Figs. 1c, d, S3). Large genomes are expected to be associated with greater phosphorus demand and slower growth than small genomes [11, 12]. In contrast, bacteria with smaller genomes have high nutrient use efficiency but reduced nutrient demand for genome replication and therefore competitive advantages to survive in nutrient-limited environments [12, 13]. Indeed, the *gcd* copy number showed a significant and positive correlation (*P* < 0.001) with genome size (Fig. 1c). In particular, MAGs affiliated with *Cyclobacteriaceae*, *Bryobacteraceae* and *Acidobacteriaceae* showed the greatest potential for solubilizing phosphate, with average *gcd* gene copy numbers of 6.00, 3.85 and 3.50, respectively (Fig. 1b). Hence, the increase in *gcd* gene copy number with genome size may be a specific adaptive trait of PSB to improve their capacity for phosphate solubilization.

We also explored whether the genetic potential of the 79 GCD-MAGs could be differentiated from that of the 393 non-GCD-MAGs. Principal coordinates analysis based on 10,799 genes revealed that among the 472 MAGs, functional profiles significantly differed between GCD-MAGs and non-GCD-MAGs (2.1% of the variance; *P* < 0.001), even though microbial phyla (phylogenetic distance) explained most of the functional variation (33.0% of the variance; *P* < 0.001; Fig. S1). To identify slight but significant differences in the genetic potential between GCD-MAGs and non-GCD-MAGs, we developed a random forest model by partitioning the 472 MAGs into training (70%) and test (30%) datasets (Fig. S4a) using the abovementioned 10,799 KEGG-queried genes as inputs. The tuned model differentiated between GCD-MAGs and non-GCD-MAGs with a prediction accuracy of 92.25%, thereby corroborating differences in genetic potential (Fig. S4b). Receiver operating characteristic curves with an area under the curve of 0.99 confirmed that the RF model performed well (Fig. S4c) [14]. We further explored whether we could identify genes that, in addition to *gcd*, have high predictive power for phosphate solubilization. Indeed, a total of 20 genes exhibited a highly increased mean square error and thus were found to occur more frequently in GCD-MAGs than in non-GCD-MAGs (Fig. S4d). Among these genes, biomarker genes are involved in carbon utilization (K00114 alcohol dehydrogenase, K05349 beta-glucosidase, K01785 aldose 1-epimerase), cell motility (K02556 chemotaxis protein MotA) and biosynthesis of enediyne antibiotics (K21162 enediyne biosynthesis protein E4).

The GCD-MAGs were significantly enriched in glycosyl hydrolase (GH) genes (*P* < 0.001; Fig. 1d). The increased genetic potential for polysaccharide breakdown and sugar metabolism in GCD*-*MAGs is reasonable from a biological perspective [10, 15]. For instance, characterization of the carbon-phosphorus exchange system between arbuscular mycorrhiza fungi and plants provided evidence that carbohydrate carbon is important for stimulating phosphate solubilization by gluconic acid, as it is produced by direct oxidation of glucose [16, 17]. Moreover, genes involved in phosphate metabolism, polysaccharide metabolism, cell motility and microbial competition were markedly enriched in GCD-MAGs (*P* < 0.05; Fig. 1e). In contrast, non-GCD-MAGs were significantly enriched in genes encoding transporters (*ABC transporters*) and in genes involved in lipid metabolism (*P* < 0.05; Fig. 1e). Noteworthily, a higher proportion of non-GCD-MAGs encoded the high-affinity phosphate-specific transport (Pst) system, while a higher proportion of GCD-MAGs encoded the phosphate-inorganic transport (Pit) system (Table S3). This may indicate that in phosphorus-limited environments, non-GCD-MAGs compete more effectively for phosphorus resources with other biota than GCD-MAGs.

To expand our knowledge of the phylogenetic diversity of PSB and to corroborate the validity of our results, we extended our analysis to a dataset of 692 genomes collected from various environments and pure cultures that represent most members of the 18 bacterial families to which our 79 GCD-MAGs belong and whose genome has been sequenced (Fig. 2a). This approach revealed a total of 229 GCD genotypes, with most being assigned to *Sphingomonadaceae* (31), *Bryobacteraceae* (30), *Chitinophagaceae* (29), *Caulobacteraceae* (25), *Cyclobacteriaceae* (15), and *Gemmatimonadaceae* (18). These GCD genotypes were assigned to 88 genera and 150 species, thereby suggesting the presence of high but yet uncharacterized PSB species-level diversity in public databases. Again, a significant correlation occurred between the *gcd* copy number and genome size (*P* < 0.001; Fig. S5). The genetic potential to produce gluconic acid was not conserved at the family or genus level but varied at the species level (Fig. 1a). The expanded genome dataset confirmed that on average, GCD genotypes significantly differed from non-GCD genotypes in terms of metabolic traits (*P* < 0.01), genome size (*P* < 0.01), GH gene ratio (*P* < 0.01), and functional diversity (*P* < 0.05) (Fig. 2b, c). The significant enrichment of particular metabolic traits in GCD genotypes, including phosphate metabolism, polysaccharide metabolism, cell motility and microbial competition, was well confirmed in the dataset of 692 genomes collected from various environments and pure cultures (*P* < 0.05; Fig. 2d). These results indeed reduce the risk of potential biases caused by phylogenetic distance (Fig. 2c, d). In addition, we validated the positive correlation between *gcd* copy number per genome and polysaccharide metabolism (fructose and mannose metabolism and starch and sucrose metabolism) (*P* < 0.05) (Fig. S5). This result was anticipated because increasing organic carbon availability in soil systems promotes microbial phosphorus solubilization [18, 19], which is known to be an energy-costing process that requires substantial metabolic investment [20]. In summary, the results of this study reveal the genome-resolved diversity of PSB in agricultural soils and enhance our understanding of their genetic composition. In particular, our research revealed a link between phosphate solubilization capacity and increased potential for polysaccharide hydrolysis and carbohydrate metabolism. This unique link corresponds strongly to the positive relationship between the population density of PSB and available dissolved organic carbon in the soil [10].

**Fig. 2:**
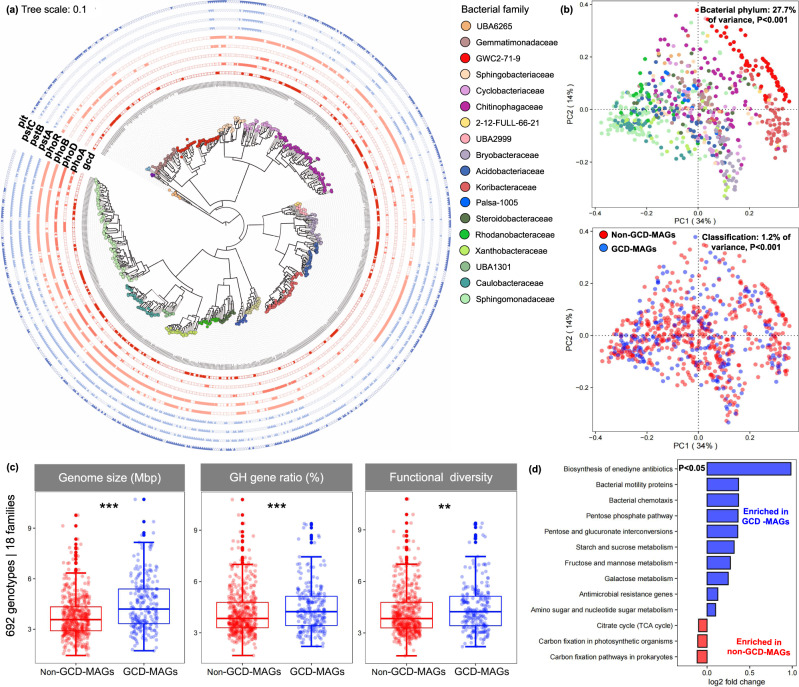
Maximum-likelihood tree of 692 genotypes (genomes from different environments and pure cultures) representing the 18 bacterial families to which one or more of our 79 GCD-MAGs were assigned. The tree thus comprises 160 MAGs obtained in this study and 532 genotypes whose sequences were downloaded from the GTDB-TK database. The tree is based on a concatenated alignment of 120 universal, single-copy marker genes in the GTDB-TK database. The copy number of *gcd* genes and the distribution of genes encoding high-affinity transporters (*pstABC*), low-affinity transporters (*pit*), components of the two-component regulatory system involved in the phosphate regulon (*phoR/phoB*), and alkaline phosphatases (*phoD* and *phoA*) among the 692 genotypes are shown (**a**). Principle coordinates analysis of genotypes that contain or do not contain *gcd*. The analysis is based on taxonomic assignment and functional profiles involving 10,799 KEGG-queried genes (**b**). Distribution range of genome size, GH gene ratio, and functional diversity between the 692 genotypes that represent the 18 bacterial families to which one or more of our 79 GCD-MAGs were assigned. Functional diversity is represented by the Shannon index and was calculated based on 10,799 KEGG-queried genes (**c**). Functional traits whose presence significantly (*P* < 0.05) differs between GCD-MAGs and non-GCD-MAGs. The analysis was performed at KEGG level 3 using DESeq2 and the family-level dataset of 692 genotypes (**d**).

## Supplementary information

Supplement information
