# Supplementary material for: Genome-resolved metagenomics identifies the particular genetic traits of phosphate-solubilizing bacteria in agricultural soil
Source: ISME Commun. 2022 Feb 16;2:17. doi: 10.1038/s43705-022-00100-z (PMC9723772; doi:10.1038/s43705-022-00100-z)
Supplement: Supplementary file 1 — Supplement information [file 43705_2022_100_MOESM1_ESM.pdf]

Supplementary information

Genome-resolved metagenomics identifies the particular genetic traits of phosphate-solubilizing bacteria in agricultural soil

Xingjie Wu[a], Zhenling Cui[a], Jingjing Peng[a*], Fusuo Zhang[a], Werner Liesack[b],

[a]College of Resources and Environmental Sciences, National Academy of Agriculture Green Development, Key Laboratory of Plant-Soil Interactions, Ministry of Education, China Agricultural University, Beijing, 100193, China.

[b]Research Group "Methanotrophic Bacteria and Environmental Genomics/Transcriptomics", Max Planck Institute for Terrestrial Microbiology, Marburg, 35043, Germany.

Contents

- Supplementary materials and methods 1
- Supplementary figures 5
- Supplementary tables 4

Supplementary references 23

* Corresponding author:

Email: jingjing.peng@cau.edu.cn (J. Peng)

Supplementary materials and methods

Sampling sites

Soil sampling was conducted from six long-term (> 10 years) field studies across China, with the agricultural management being based on chemical fertilization: (1) BD (N38°78, E115°28), (2) QZ (N36°78, E114°94), (3) GZL (N43°50, E124°82), (4) HEB (N45°55, E126°95), (5) LS (N43°31, E124°33) and (6) TY (N37°94, E112°48) (Table S1). These sites were managed with wheat-maize rotation system using long-term fertilization practices, including both conventional and optimal fertilization [1]. Soil samples were collected after maize harvest from July to October 2020. Each fertilizer treatment involved four replicates. For each replicate, five soil cores were taken from 0-30 cm depth and combined to one composite sample, thereby resulting in a total of 80 samples (Table S1). After careful removal of rocks, soil samples were transferred into 2 ml sterilized microcentrifuge tubes and stored at -20 ^{o}C until DNA extraction.

DNA extraction and metagenomic sequencing

Total genomic DNA was extracted from each soil sample using the FastDNA Spin Kit (MP Biochemicals, LLC) following the manufacturer's protocol. The DNA extracts were purified by the Wizard DNA cleanup system (Axygen Bio, USA) according to manufacturer's recommendations, resuspended in nuclease-free water, and stored at 4 °C. Quality and concentration of DNA extracts were checked using Qubit Fluorometer (Invitrogen, California, USA). Using the Agilent 2100 Bioanalyzer

(Agilent Technologies, Palo Alto, CA), fragment lengths of 300-500 bp were selected for preparation of the 80 metagenomic libraries. The Illumina NextSeq platform with 2×150 bp reads was used for sequencing. Ten Gb (gigabases) of raw sequence data were on average obtained for each of the 80 samples (total of approximately 800 Gb).

Metagenomic binning

We used a previously described analysis pipeline for metagenomic binning [2-4]. In brief, raw reads with artifacts, low-quality reads and unpaired reads (quality score less than 20) were filtered in Trimmomatic (Version 0.35; [5]). The pre-processed reads of the four replicate datasets obtained for each treatment were co-assembled into contigs by MEGAHIT (Version 1.1.3) with *kmer* values ranging from 21 to 121 [6]. Contigs and clean data were binned using the MetaWRAP pipeline with default parameters [7]. Completeness, contamination, genome size, and GC content of output MAGs were evaluated by CheckM (Version 1.1.2) with the following parameter values: "lineage_wf, -t 16, -x fa" [8]. A total of 472 MAGs with a quality index (defined as completeness – 5× contamination) greater than 45 were selected for further analysis [9, 10]. Taxonomic classification of the MAGs was performed by searching the GTDB-TK (Version 1.3.0) database in classify_wf mode [11]. Protein-coding genes were identified with Prokka (Version 1.14.6) by predicting for each MAG in default single genome mode [12]. Predicted open reading frames were aligned against the NCBI-nr database by DIAMOND in blastp mode with parameters of "-k 5 -e 1e-4 -- sensitive" [13]. Functional annotation of the MAGs was carried out by using the Kyoto Encyclopedia of Genes and Genomes (KEGG) database in the

MAGAN6 Ultimate Edition with default parameters [14, 15]. Genes encoding carbohydrate active enzymes (CAZymes) were queried against CAZyme database by Diamond (Version 0.9.24.125) with an e-value of 10^{-5} [13]. Maximum-likelihood phylogenetic trees were constructed for a concatenate of 120 universal core gene markers in GTDB-TK (gtdbtk de_novo_wf mode). The tree files were visualized by the Interactive Tree of Life (iTOL; Version 5.0) platform [16].

Identification of gcd genes

The *gcd* gene sequences in each MAG were queried against KEGG database with the annotation hits to K00117 (quinoprotein glucose dehydrogenase [EC:1.1.5.2]). Gene-derived amino acid sequences of soluble GCD (EC 1.1.99.35) were identified by InterProScan [17, 18] (Table S2). Soluble GCD is not involved in inorganic phosphate solubilization and therefore was excluded from further analysis of *gcd*-containing MAGs. We created a maximum-likelihood tree of membrane-bound GCD (EC.1.1.5.2), in which we used sequences of soluble GCD identified in our MAGs and, in addition, in genomes downloaded from NCBI as an outgroup. The GCD sequences were automatically aligned using MUSCLE in MEGA with default settings [19] and manually inspected. The tree file was visualized by the online iTol platform (Interactive Tree of Life online interface). The maximum-likelihood tree further confirmed that three of the 472 MAGs (one MAG affiliated with *Nitrospirae* and another two MAGs affiliated with *Planctomycetes*) contained soluble GCD.

Machine learning approach

A random forest model was applied to distinguish between GCD-MAGs and non-GCD-MAGs using a dataset of 472 bacterial MAGs and a gene matrix consisting of 10,799 KEGG genes. Random forest has been widely applied in microbial research as it is able to reveal subtle differences between features [20, 21]. Model training and validation was performed by randomly partitioning the datasets to 70% of training data and 30% of test data using the *randomForest* R package. The model was trained by using the training datasets, and the predictivetable s4 accuracy was evaluated by the testing datasets. The random forest analysis was performed with the number of trees being set to 1000. Receiver operating characteristic (ROC) curves and the area under the ROC curve (AUC) were used to evaluate the performance of the random forest model. The prediction power of each KEGG level 4 gene was assessed based on mean square error.

Statistical analysis

The statistical analysis was implemented in R environment (Version 3.6.1). Significant difference was tested by analysis of variance after normalization and homogeneity test. *P*-values were corrected for the false discovery rate by the method of Benjamini and Hochberg [22]. To identify functional genes whose presence significantly varied between MAGs, we applied a differentially abundant gene analysis in the R package (*DESeq2*) [23]. Principle coordinates analysis (PCoA) using unweighted UniFrac distance and permutational multivariate analysis of variance

(PERMANOVA) were used to analyze the functional variation between MAGs based on KEGG level 4 in the R package *vegan*. The abundance matrix of 10,799 KEGG genes was taken as the KEGG profile to perform PCoA. The obtained 472 MAGs were deposited in the National Center for Biotechnology Information (NCBI) with the accession number PRJNA744024.

GenBank downloads

A total of 532 genome sequences were downloaded from GenBank. These were affiliated with those 18 bacterial families to which our 79 GCD-MAGs were assigned. The 160 MAGs obtained in our study and affiliated with these 18 families were additionally used to validate the link between *gcd* presence and particular genetic traits in phosphate-solubilizing bacteria. Thus, a total dataset of 692 genotypes was used for validation analysis. Using the above-mentioned pipeline (Prokka, NCBI-nr, CAZyme, KEGG and GTDB-TK) and *gcd* identification approach (InterProScan and maximum-likelihood tree), we were able to separate this dataset into 229 GCD genotypes and 463 non-GCD genotypes that span 18 bacterial families.

Supplementary figures

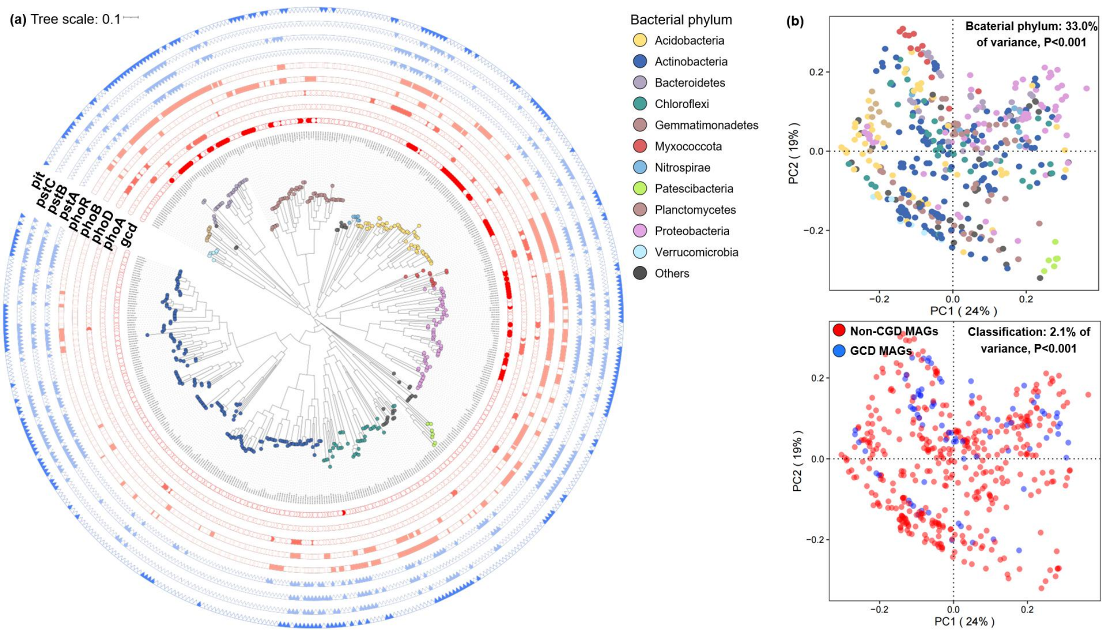

Fig. S1 Maximum-likelihood tree of 472 MAGs with good overall quality based on a concatenated alignment of 120 universal, single-copy marker genes in GTDB-TK. The genome size of the 472 MAGs ranged from 0.538 to 9.440 Mb. Their taxonomic affiliation spanned 21 bacterial phyla, with the greatest number of MAGs affiliated with *Actinobacteria* (153), *Proteobacteria* (66), *Acidobacteria* (54), *Gemmatimonadetes* (54), and *Chloroflexi* (41). The MAGs assigned to miscellaneous bacterial phyla (e.g., *Binatota*, *Eremiobacteria, Fibrobacteria*) were grouped as 'others'. Dots, rectangle and triangle indicate genes involved in P-cycling (a). Principle coordinates analysis of MAGs that contain or do not contain *gcd*. The analysis is based on functional profiles using 10,799 genes (KEGG level 4) (b).

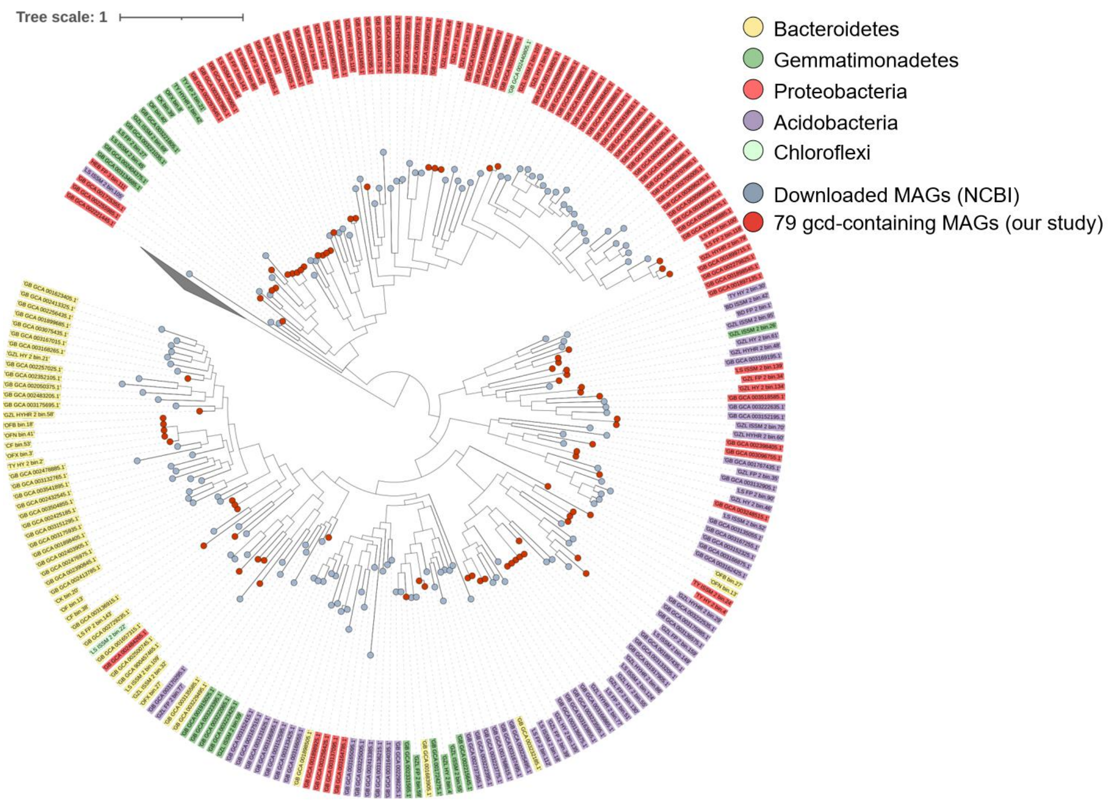

Fig. S2 Maximum-likelihood tree of *gcd* genes identified in our 79 GCD-MAGs (red dots). These are shown in relation to *gcd* genes identified in reference genomes that were downloaded from NCBI and represent most members of the 18 bacterial families to which our 79 GCD-MAGs belong and whose genome has been sequenced (blue dots). The tree was constructed on the inferred amino acid sequences of membrane-bound GCD (EC 1.1.5.2). The root of the tree was determined by sequences of soluble GCD (EC 1.1.99.35) identified in our MAGs and, in addition, in genomes downloaded from NCBI.

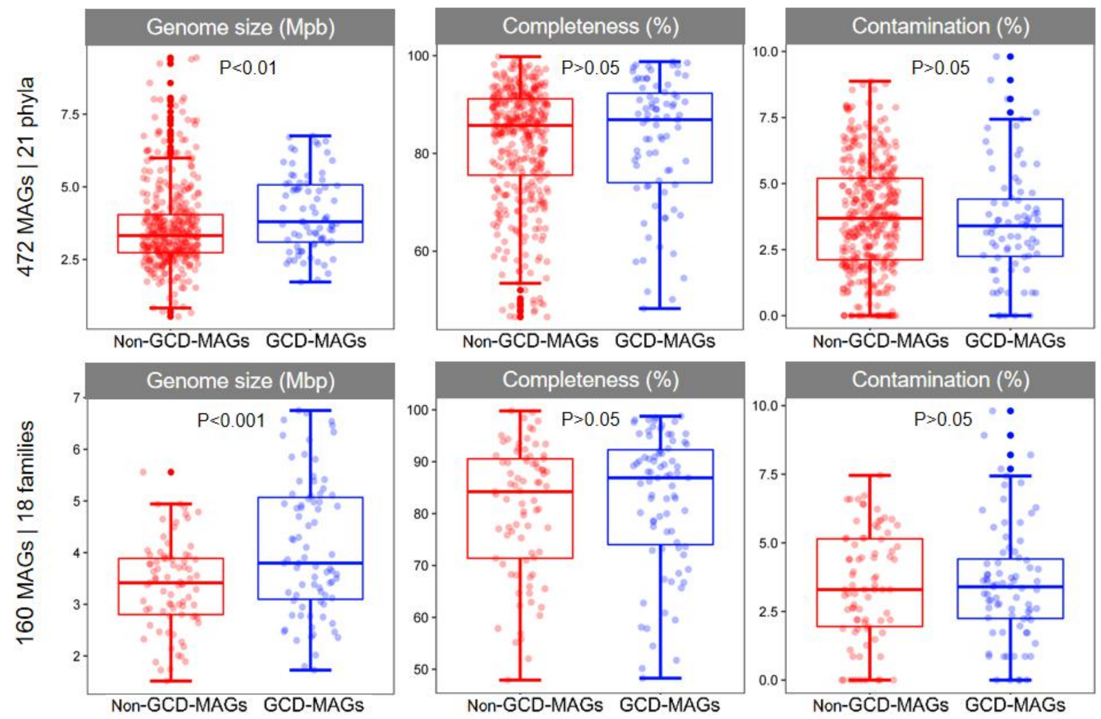

Fig. S3 Genome size, completeness, and contamination of 472 good overall quality MAGs obtained in this study and a subset of 160 MAGs covering the 18 families to which the GCD-MAGs belong. The GCD-MAGs exhibit, on average, significantly ($P < 0.05$) larger genomes than the MAGs that do not contain *gcd*. Completeness and contamination among the 472 MAGs were on average 82.2% and 3.7%, respectively. The subset of 160 MAGs was comprised of MAGs than contain (79 MAGs) or do not contain (81 MAGs) the *gcd* gene. There was no significant difference ($P > 0.05$) in completeness and contamination between GCD-MAGs and non-GCD-MAGs.

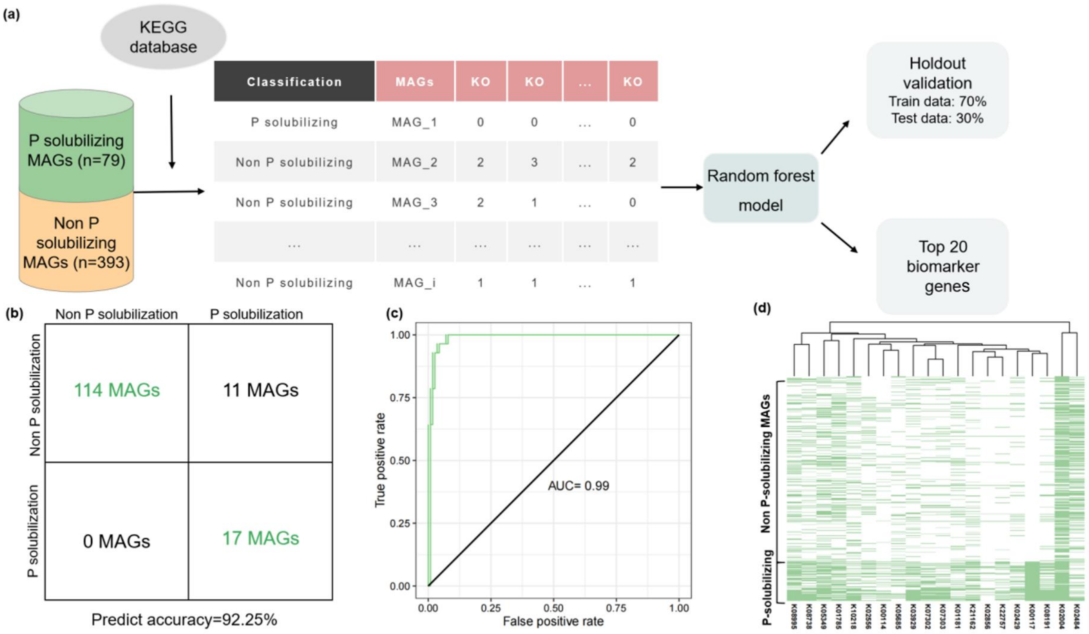

Fig. S4 Machine learning prediction of MAGs or genomes that have or do not have the genetic potential for P solubilization (a). Accuracy of the random forest model (b). Receiver operating characteristic (ROC) curves and the area under the ROC curve (AUC) to evaluate model performance (c). The top 20 most important predictors are shown (d).

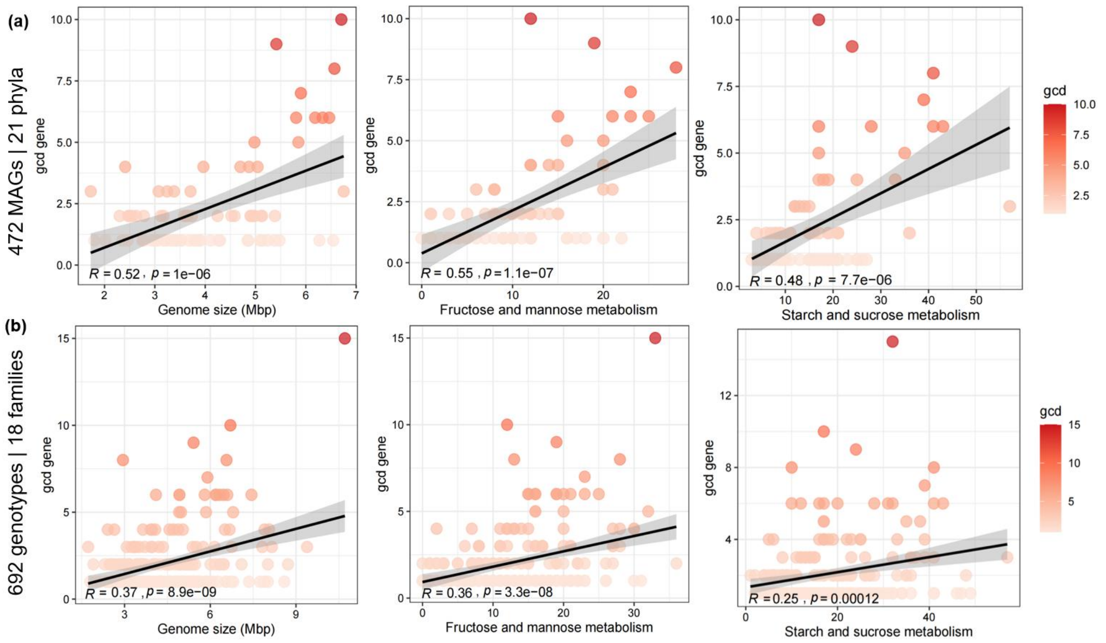

Fig. S5 Correlations of *gcd* copy number per genome with genome size and particular genetic traits (fructose and mannose metabolism; starch and sucrose metabolism). The correlation analysis is based on the 79 GCD-MAGs obtained in this study (a) and 229 family-level GCD genotypes (b).

Table S1 GeoCoordinates of the six sampling sites (field studies)

Name	Site	Province	Location	Samples[1]	MAGs[2]
QZ	QuZhou	Hebei	N36°78 E114°94	24 (6)[3]	64
BD	BaoDing	Hebei	N38°78 E115°28	8 (2)[3]	21
TY	TaiYuan	ShanXi	N37°94 E112°48	16 (4)[3]	60
GZL	GongZhuLing	JiLin	N43°50 E124°82	16 (4)[3]	196
LS	LiShu	JiLin	N43°31 E124°33	8 (2)[3]	112
HEB	HaErBin	HeiLongJiang	N45°55 E126°95	8 (2)[3]	19

[1]A total of 80 samples were used for metagenomic analysis.
[2]Total number of high- and medium-quality MAGs obtained.
[3]Number of treatments in each sampling site is indicated in parenthesis.

Table S2 Identification of gene-inferred soluble GCD proteins by InterProScan.

Name	Length	*P*-value	Protein
OF_bin.2	324	1.03E-26	Quinoprotein alcohol dehydrogenase-like superfamily
GB_GCA_002292165.1	265	-	-
GZL_ISSM_2_bin.79	1115	4.05E-20	Soluble quinoprotein glucose/sorbosone dehydrogenase
GZL_FP_2_bin.61	1106	1.70E-21	Soluble quinoprotein glucose/sorbosone dehydrogenase
GB_GCA_003223455.1	1159	2.62E-12	Soluble quinoprotein glucose/sorbosone dehydrogenase
GB_GCA_003286915.1	1134	4.45E-11	Soluble quinoprotein glucose/sorbosone dehydrogenase
GB_GCA_003537495.1	1124	4.97E-09	Soluble quinoprotein glucose/sorbosone dehydrogenase
GB_GCA_002352225.1	1125	5.62E-09	Soluble quinoprotein glucose/sorbosone dehydrogenase
GB_GCA_001567185.1	1107	1.06E-06	Soluble quinoprotein glucose/sorbosone dehydrogenase
GB_GCA_002471275.1	1130	1.57E-12	Soluble quinoprotein glucose/sorbosone dehydrogenase
GB_GCA_002292365.1	1117	1.18E-06	Soluble quinoprotein glucose/sorbosone dehydrogenase
GB_GCA_003242315.1	1136	2.09E-11	Soluble quinoprotein glucose/sorbosone dehydrogenase
GB_GCA_002691385.1	1107	1.14E-06	Soluble quinoprotein glucose/sorbosone dehydrogenase
GB_GCA_002470835.1	1129	11.444027	Cytochrome c-like domain

Table S3 Total number of GCD-MAGs and non-GCD-MAGs that harbor particular genes involved in P-cycling.

**Function**	**Gene[1]**	**Names**	**KO**	**79 GCD-MAGs**	**393 Non-GCD-MAGs**
Inogranic P solubilization	*gcd[1]*	PQQGDH (quinoprotein glucose dehydrogenase)	K00117	79 (100)[2]	0
Organic P mineralization	*phoA*	alkaline phosphatase (PhoA)	K01077	6 (7.6)	16 (4.1)
Organic P mineralization	*phoD*	alkaline phosphatase (PhoD)	K01113	5 (6.3)	52 (13.2)
Regulatory gene	*phoR*	phosphate regulon sensor histidine kinase (PhoR)	K07636	37 (46.8)	126 (32.0)
Regulatory gene	*phoB*	phosphate regulon response regulator PhoB	K07657	26 (32.9)	97 (24.7)
Transporter	*pit*	phosphate inorganic transporter	K03306	44 (55.7)	153 (38.9)
Transporter	*pstA*	phosphate-specific transport system subunit PstA	K02038	19 (24.1)	138 (35.1)
Transporter	*pstB*	phosphate-specific transport system subunit PstB	K02036	21 (26.6)	135 (34.4)
Transporter	*pstC*	phosphate-specific transport system subunit PstC	K02037	16 (20.3)	136 (34.6)

[1]These genes were shown to be involved in phosphorus metabolism [18].

[2] The percentage proportion of genes detected in GCD-MAGs and non-GCD-MAGs, respectively.

Table S4 Percentage proportion of GCD genotypes on total genotypes in each of the 18 bacterial families analyzed and the average *gcd* gene copy number in GCD genotypes of the respective family. The analysis is based on a total dataset of 692 genotypes.

Family	Total number of genotypes	Number of GCD genotypes	Percentage proportion	Average *gcd* gene copy number
*Bryobacteraceae**	39	30	76.92%	3.67
*Cyclobacteriaceae**	39	24	61.54%	3.27
Alphaproteobacteria (*UBA1301*)	13	6	46.15%	3.17
Sphingobacteriaceae	31	7	22.58%	2.71
Acidobacteriaceae	44	14	31.82%	2.21
Chitinophagaceae	90	29	32.22%	2.07
Xanthobacteraceae	31	4	12.90%	2.00
Vicinamibacterales (*2-12-FULL-66-21*)	5	2	40.00%	2.00
Sphingomonadaceae	105	31	29.52%	1.87
Steroidobacteraceae	20	6	30.00%	1.83
Vicinamibacterales (*UBA2999*)	15	11	73.33%	1.73
Rhodanobacteraceae	24	11	45.83%	1.36
Gemmatimonadaceae	41	18	43.90%	1.33
Gemmatimonadales (*GWC2-71-9*)	59	6	10.17%	1.33
Caulobacteraceae	46	25	54.35%	1.20
Koribacteraceae	68	10	14.71%	1.00
Thermomicrobiales (*UBA6265*)	8	2	25.00%	1.00
Burkholderiales (*Palsa-1005*)	14	2	14.29%	1.00

*Most members of the *Bryobacteraceae* (76.9%) and *Cyclobacteriaceae* (61.5%) possess the genetic potential to produce gluconic acid.

References

1. Cui Z, Zhang H, Chen X, Zhang C, Ma W, Huang C, et al. Pursuing sustainable productivity with millions of smallholder farmers. Nature. 2018;555:363-366.
2. Wu X, Peng J, Liu P, Bei Q, Rensing C, Li Y, et al. Metagenomic insights into nitrogen and phosphorus cycling at the soil aggregate scale driven by organic material amendments. Sci Total Environ. 2021;785:147329.
3. Wu X, Liu P, Wegner CE, Luo Y, Xiao KQ, Cui Z, et al. Deciphering microbial mechanisms underlying soil organic carbon storage in a wheat-maize rotation system. Sci Total Environ. 2021;788, 147798.
4. Wu X, Rensing C, Han D, Xiao KQ, Dai Y, Tang Z, et al. Genome-resolved metagenomics reveals distinct phosphorus acquisition strategies between soil microbiomes. mSystems. 2022; 7(1): e01107-21.
5. Bolger AM, Lohse M, Usadel B. Trimmomatic: a flexible trimmer for Illumina sequence data. Bioinformatics. 2014;30:2114-20.
6. Li D, Liu C, Luo R, Sadakane K, Lam T. MEGAHIT: an ultra-fast single-node solution for large and complex metagenomics assembly via succinct de Bruijn graph. Bioinformatics. 2015;31:1674-1676.
7. Uritskiy GV, DiRuggiero J, Taylor J. MetaWRAP - a fexible pipeline for genome-resolved metagenomic data analysis. Microbiome. 2018;6:158.
8. Parks DH, Imelfort M, Skennerton CT, Hugenholtz P, Tyson GW. CheckM: assessing the quality of microbial genomes recovered from isolates, single cells, and metagenomes. Genome Res. 2015;25:1043-1055.
9. Bowers RM, Kyrpides NC, Stepanauskas R, Harmon-Smith M, Doud D, Reddy TBK, et al. Minimum information about a single amplified genome (MISAG) and a metagenome-assembled genome (MIMAG) of bacteria and archaea. Nat Biotechnol. 2017;35:725-31.

10. Lin Y, Wang L, Xu K, Li K, Ren H. Revealing taxon-specific heavy metal-resistance mechanisms in denitrifying phosphorus removal sludge using genome-centric metaproteomics. Microbiome. 2021;9.
11. Chaumeil PA, Mussig AJ, Hugenholtz P, Parks DH. GTDB-Tk: a toolkit to classify genomes with the Genome Taxonomy Database. Bioinformatics. 2020;36:1925-1927.
12. Seemann T. Prokka: rapid prokaryotic genome annotation. Bioinformatics. 2014;30:2068-9.
13. Buchfink B, Xie C, Huson, DH. Fast and sensitive protein alignment using DIAMOND. Nat Methods. 2014;12:59-60.
14. Huson DH, Mitra S, Ruscheweyh, HJ, Weber, N, Schuster, SC. Integrative analysis of environmental sequences using MEGAN4. Genome Res. 2011;21:1552-1560.
15. Kanehisa M, Sato Y, Morishima K. BlastKOALA and GhostKOALA: KEGG tools for functional characterization of genome and metagenome sequences. J Mol Biol. 2016;428:726-31.
16. Letunic I, Bork P. Interactive Tree Of Life (iTOL) v4: recent updates and new developments. Nucleic Acids Res. 2019;47:256-9.
17. Jones P, Binns D, Chang HY, Fraser M, Li W, McAnulla C, et al. InterProScan 5: genome-scale protein function classification. Bioinformatics. 2014;30:1236-40.
18. Liang J, Liu J, Jia P, Yang T, Zeng Q, Zhang S, et al. Novel phosphate-solubilizing bacteria enhance soil phosphorus cycling following ecological restoration of land degraded by mining. ISME J. 2020;14:1600-1613.
19. Tamura K, Peterson D, Peterson N, Stecher G, Nei M, Kumar S. MEGA5: Molecular evolutionary genetics analysis using maximum likelihood, evolutionary distance, and maximum parsimony methods. Mol Biol Evol. 2011;28:2731-2739.

20. Chang H, Haudenshield JS, Bowen CR, Hartman GL. Metagenome-wide association study and machine learning prediction of bulk soil microbiome and crop productivity. Front Microbiol. 2017;8.
21. Ye L, Mei R, Liu WT, Ren H, Zhang XX. Machine learning-aided analyses of thousands of draft genomes reveal specific features of activated sludge processes. Microbiome. 2020;8.
22. Benjamini Y, Hochberg Y. Controlling the false discovery rate: a practical and powerful approach to multiple testing. J R Stat Soc Ser B. 1995;57:289-300.
23. Love MI, Huber W, Anders S. Moderated estimation of fold change and dispersion for RNA-seq data with DESeq2. Genome Biol. 2014;15(12):1-21.
